# Brain and Head-and-Neck MRI in Immobilization Mask: A Practical Solution for MR-Only Radiotherapy

**DOI:** 10.3389/fonc.2019.00647

**Published:** 2019-07-17

**Authors:** Stefano Mandija, Federico D'Agata, Robin J. M. Navest, Alessandro Sbrizzi, Rob H. N. Tijssen, Marielle E. P. Philippens, Cornelis P. J. Raaijmakers, Enrica Seravalli, Joost J. C. Verhoeff, Jan J. W. Lagendijk, Cornelis A. T. van den Berg

**Affiliations:** ^1^Computational Imaging Group for MRI Diagnostics and Therapy, Center for Image Sciences, University Medical Center Utrecht, Utrecht, Netherlands; ^2^Department of Radiotherapy, University Medical Center Utrecht, Utrecht, Netherlands; ^3^Department of Neurosciences, University of Turin, Turin, Italy

**Keywords:** brain, head-and-neck, immobilization mask, MR-only, radiotherapy

## Abstract

In brain/head-and-neck radiotherapy (RT), thermoplastic immobilization masks guarantee reproducible patient positioning in treatment position between MRI, CT, and irradiation. Since immobilization masks do not fit in the diagnostic MR head/head-and-neck coils, flexible surface coils are used for MRI imaging in clinical practice. These coils are placed around the head/neck, in contact with the immobilization masks. However, the positioning of these flexible coils is technician dependent, thus leading to poor image reproducibility. Additionally, flexible surface coils have an inferior signal-to-noise-ratio (SNR) compared to diagnostic coils. The aim of this work was to create a new immobilization setup which fits into the diagnostic MR coils in order to enhance MR image quality and reproducibility. For this purpose, a practical immobilization setup was constructed. The performances of the standard clinical and the proposed setups were compared with four tests: SNR, image quality, motion restriction, and reproducibility of inter-fraction subject positioning. The new immobilization setup resulted in 3.4 times higher SNR values on average than the standard setup, except directly below the flexible surface coils where similar SNR was observed. Overall, the image quality was superior for brain/head-and-neck images acquired with the proposed RT setup. Comparable motion restriction in feet-head/left-right directions (maximum motion ≈1 mm) and comparable inter-fraction repositioning accuracy (mean inter-fraction movement 1 ± 0.5 mm) were observed for the standard and the new setup.

## Introduction

In daily radiotherapy (RT) clinical practice, Magnetic Resonance Imaging (MRI) is a principal image modality (1, 2). Compared to Computed Tomography (CT), MRI offers superior soft tissue contrast, which is crucial for visualization and delineation of tumors and organs at risk and for monitoring of RT treatments (3, 4).

An essential requirement in RT is the reproducibility of patient positioning between MRI, CT, and irradiation fractions (5). For this purpose, immobilization masks are currently used for brain and head-and-neck RT (6). The use of immobilization masks ensures that the MRI and CT exams are performed in the same treatment position (TP). Although this allows reproducible patient positioning, minimization of registration errors, and guarantee correct dose calculation, it also leads to several disadvantages from an MRI imaging perspective.

First, a flat MRI table top with customized anchor points is needed to replicate the same setup used during RT treatments. The replacement of the standard curved MRI table top with the flat table top requires extra preparation time.

Second, and even more important, immobilization masks are incompatible with clinically diagnostic MRI Head/Head-and-Neck (H/HN) coils, since these coils, which consist of numerous receive channels placed into a cylindrical plastic holder, only allow narrow access to a human head. For this reason, MRI images are acquired with flexible loop surface coils in RT clinical practice (7–12). Although flexible loop surface coils facilitate scanning with mask in TP, they are known to have suboptimal signal-to-noise-ratio (SNR) due to the limited number of receive channels (13, 14), compared to clinical H/HN coils. This is an evident limitation in SNR demanding MRI acquisitions, such as Fluid Attenuated Inversion Recovery imaging (FLAIR) (15). Furthermore, the positioning of surface coils is MRI technician dependent. These factors limit proper image quality to a small region in close proximity to the surface loops and compromises image reproducibility.

These latter disadvantages are even more important for an MR-only radiotherapy workflow (16). MR-only is an emerging RT treatment approach where dose calculation is performed on so-called synthetic CT images generated from MR images. In such as workflow, MRI scanning needs to be performed in treatment position. Therefore, it would be highly beneficial to have a setup that allows MRI scanning in TP with diagnostic image quality.

In this work, we explore the feasibility of a practical solution consisting of an immobilization setup redesigned to fit into diagnostic H/HN coils. We investigate and compare SNR, image quality, motion restriction and reposition accuracy of the proposed setups with respect to the standard used in daily brain and head-and-neck RT. We will demonstrate that the proposed solution allows not only comparable motion restriction and reproducibility of subject positioning to the standard RT setup, but it also allows diagnostic image quality in TP, which is not achievable in standard RT practice due to the use of flexible loop surface coils.

## Materials and Methods

### Setup Description

**Figure 1:**
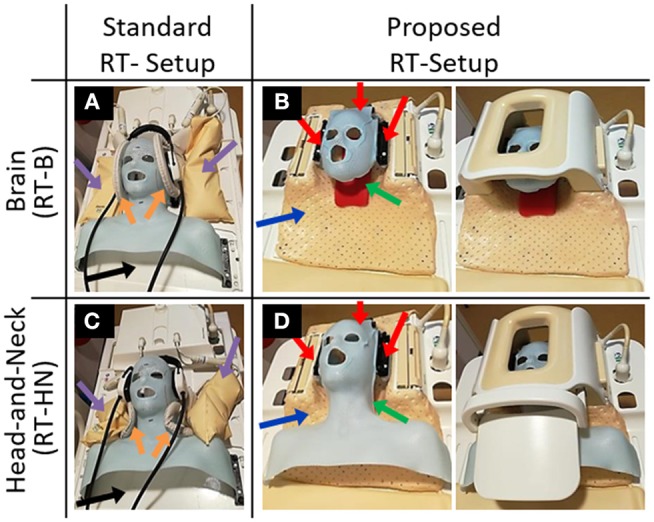
Standard and proposed setups for MR imaging in treatment position for brain **(A,B)** and head-and-neck **(C,D)** radiotherapy patients. The blue arrows indicate the newly designed thermoplastic holder. The red arrows indicate the anchor points of the immobilization masks. The green arrows indicate the neck support, which can be made patient-specific.

Four setups were adopted (Figure 1):

- The standard RT brain (RT-B) and the standard head-and-neck (RT-HN) setups consisting of an individualized five-points head-and-shoulder mask (Posicast; Civco Sinmed, Reeuwijk, The Netherlands) fixated to an in-house developed base plate (Figure 1, black arrows), which was positioned on the MRI flat table top. This base plate also accommodated the standard neck support. For MR imaging, two pairs of flexible loop surface coils were placed on the lateral sides of the brain/head-and-neck immobilization mask (15/20 cm diameter, respectively for RT-HN and RT-B, Figure 1, orange arrows) and fixated using sand bags (Figure 1, purple arrows);
- The proposed RT-B setup consisting of an individualized three-points head-only mask inside the MRI clinical H coil, and the proposed RT-HN setup consisting of a three-points head mask extended to the shoulders inside the MRI clinical HN coil. To fit the new immobilization prototype into the H/HN coils, a new thermoplastic mask holder was created (Figure 1, blue arrow). This holder was designed to perfectly fit the base of the H/HN coils and to host the anchor points for the immobilization masks (Figure 1, red arrows). This holder can also be used for both 1.5T and 3T MRI scanners given the same geometry of the base for these clinical H/HN coils. The same neck support used in the standard reference setups was adopted (Figure 1, green arrow).

### MRI Tests

MR images of 2 volunteers (1 male 30 years old, 1 female 27 years old) were acquired using a 3T Ingenia MRI (Philips Healthcare, Best, The Netherlands), after obtaining written inform consent. The analyses were carried out in Matlab R2015b (The Mathworks, Natick, Massachusetts, USA). Several comparisons were performed: SNR, image quality, movement restriction, and inter-fraction subject repositioning. The list of the performed MRI sequences and the adopted MRI sequence parameters are reported in the Supplementary Table 1.

#### SNR Test and Examples of Image Quality

The SNR levels obtained with the standard and the proposed RT-B and RT-HN were compared. For this purpose, a 2D T1-weighted Incoherent Gradient Echo (RF spoiled) sequence was acquired for each setup (2 dynamics, the second being a noise-only scan). From the acquired images, SNR maps were computed according to Kellman and McVeigh (17).

Afterwards, MR images were acquired as examples of the impact of the different SNR levels obtained with the different setups. For this purpose, the same MRI sequences were acquired using the standard and proposed setups, thus allowing direct image comparison. In particular, 3 standard clinical sequences (3D T1-weighted Ultrafast Gradient Echo, 3D T2-weighted Turbo Spin Echo FLAIR and 2D T2-weighted Turbo Spin Echo) were acquired for the standard and the proposed RT-B. For RT-HN, 2 standard clinical sequences were acquired (2D T1-weighted Turbo Spin Echo and 2D T2-weighted Turbo Spin Echo mDIXON).

#### Movement Restriction Test

We investigated the maximum movement possible for one volunteer in the sagittal and transversal planes for the different setups. The male subject was asked to rotate the head: 1) in the feet-head direction (motion in the MR sagittal plane, head rotation around the left-right body axis), 2) in the left-right direction (motion in the MR transverse plane, head rotation around the cranio-caudal body axis) during cine-MRI acquisitions. As shown in Houweling et al. (18), the largest rotation angles are generally observed for head rotations around these two axis. For this reason, we asked the subject to rotate the head as aforementioned as much as possible in order to simulate the worst-case scenarios. Separate cine-MRI acquisitions were performed for each of the two directions (sagittal and transversal) using a single slice T1-weighted balanced Gradient Echo sequence (300 frames, about 1.5 min).

To quantify the movement in time, we used the Optical Flow algorithm implemented in the RealTITracker toolbox (19). First, 2D displacement vectors were computed between each cine-MRI frame for each voxel. Then, the magnitude of these vectors was calculated voxel-wise, leading to absolute displacement maps. Finally, for each map representing one time step, the average absolute value of the displacement was calculated as the mean of the absolute displacement values of each voxel in the head, excluding the background (air). This allowed us to evaluate the time evolution of the average absolute movement.

#### Inter-fraction Repositioning Test

To investigate the accuracy in inter-fraction subject repositioning, an MRI sequence was acquired twice on the female subject for each setup. Between MRI acquisitions, the immobilization mask was removed and the subject was asked to get off the MRI table.

For brain, two high resolution 3D T1-weighted Ultrafast Gradient Echo sequences were acquired using the standard and the proposed RT-B, respectively.

For HN, we evaluated the repositioning accuracy for the standard RT-HN, the proposed RT-B and the proposed RT-HN. The latter two were used to investigate the need of constructing a mask extended over the shoulders for HN MRI instead of a head-only mask (Figure 1, middle figures). Two high resolution 3D T1-weighted Ultrafast Gradient Echo sequences were acquired for this purpose.

The 3D displacement fields between each pair of MR acquisitions were computed using RealTITracker. From these maps, the mean and the standard deviation of the absolute displacement of the subject between the two MRI acquisitions were computed as a proxy of the reproducibility of inter-fraction subject repositioning. Using the rigid body registration module in SPM12 (https://www.fil.ion.ucl.ac.uk/spm), we finally calculated the 6 global roto-translation parameters needed to coregister the pair of scans (20).

## Results

### SNR Test and Examples of Image Quality

**Figure 2:**
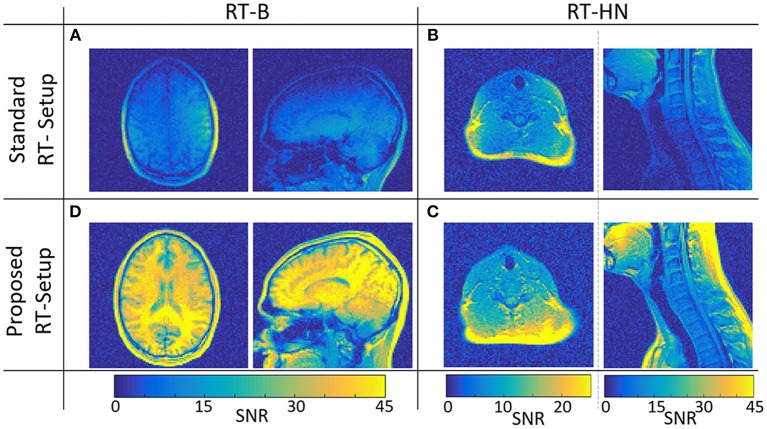
SNR maps for the standard **(A,B)** and the proposed **(C,D)** RT-B and RT-HN setups, respectively.

In Figure 2, SNR maps in transverse and sagittal orientations are shown for the standard and the proposed RT-B and RT-HN.

The SNR achieved using the proposed RT-B was on average 4.1 times higher compared to the SNR of the standard setup, which was particularly low in regions far away from the surface loop coils, e.g., the high convexity and the frontal regions.

For the standard RT-HN, the SNR was highly dependent on the spatial position. Only locally, just below the flex coils, the SNR was comparable to the proposed RT-HN (see transverse images). This was not the case for more deeply located anatomical regions, e.g., throat, chest, and shoulders (sagittal images), where the SNR of the proposed RT-HN was on average 2.8 times higher.

**Figure 3:**
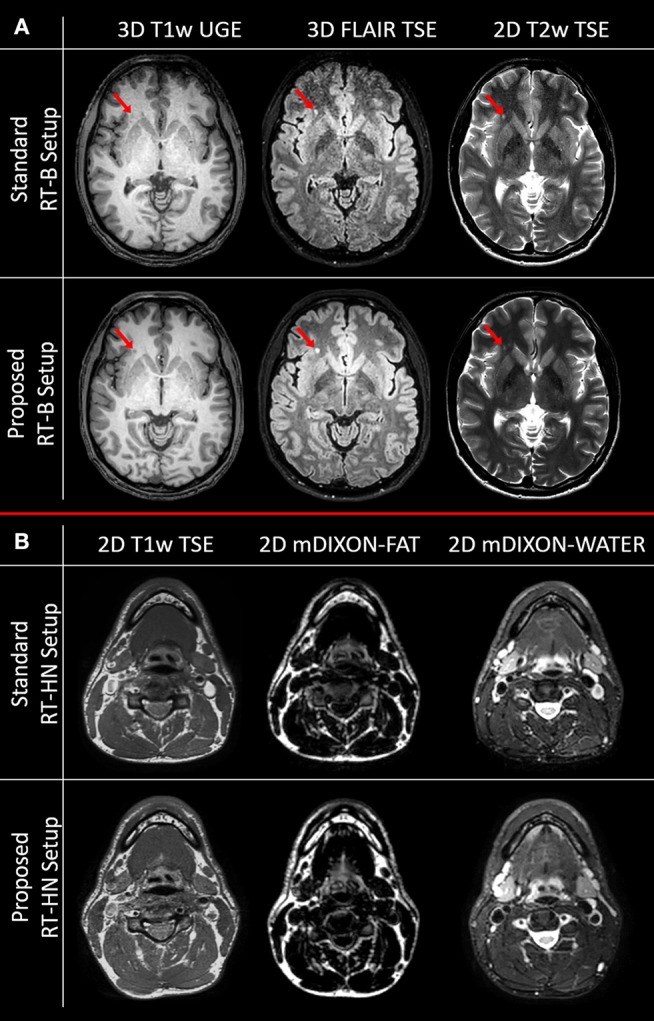
Image quality comparison for clinically acquired brain **(A)** and head-and-neck **(B)** MRI sequences for the standard and the proposed setups. The red arrows indicate a white matter lesion.

In Figure 3, the images acquired with the brain and HN MRI scans listed in section SNR test and examples of image quality are shown.

As predicted by the SNR maps, the brain images acquired with the standard RT-B had an inferior image quality compared to the brain images acquired with the proposed RT-B. This was evident in the frontal area where the low SNR led to lesions being less detectable, e.g., the white matter lesion in the frontal region (red arrows). The loss of image quality was particularly relevant for the FLAIR image.

For HN, the image quality obtained with the standard and the proposed setups was comparable in the regions below the flex coils. However, as shown in the SNR maps, the image quality is expected to be reduced for the standard RT-HN in regions away from the flex coils (e.g., low neck and shoulders).

### Movement Restriction Test

**Figure 4:**
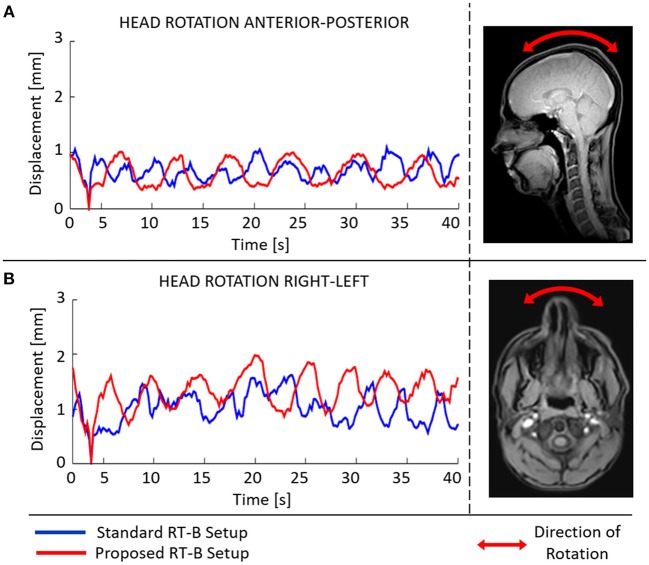
Mean absolute displacement [anterior-posterior **(A)**, right-left **(B)**] inside the radiotherapy immobilization mask as determined from cine-MRI images acquired using the standard (blue) and the proposed (red) RT-B.

In Figure 4, the capability of movement restriction is shown for the standard and the proposed RT-B. These results show that the motion restriction was comparable among setups and rarely exceed 1.5 mm.

### Inter-fraction Repositioning Test

In Table 1, the roto-translation parameters between scans and the mean/standard deviation values of the absolute displacement are reported. In the Supplementary Figures 1, 2, the inter-fraction reproducibility of subject positioning is shown for each setup by means of absolute displacement maps.

**Table 1:** Inter-fraction subject repositioning accuracy for the standard and the proposed RT-B (upper rows) and RT-HN (lower rows) setups.

		**Roto-translation^**a**^**						**Displacement^**b**^**
**Setup**		**RL** **[mm]**	**AP** **[mm]**	**FH** **[mm]**	**Pitch** **[^**°**^]**	**Roll** **[^**°**^]**	**Yaw** **[^**°**^]**	**Mean (SD)** **[mm]**
Brain	Standard RT-B	0.5	0.1	0.1	0.17	0.11	0.53	0.9 (0.5)
	Proposed RT-B	0.2	0.1	1.1	0.35	0.21	0.17	1.3 (0.4)
Head and Neck	Standard RT-HN	0.2	0.1	0.1	0.02	0.11	0.12	0.9 (0.5)
	Proposed RT-B	0.2	4.5	2.8	0.91	0.25	0.25	3.3 (1.1)
	Proposed RT-HN	0.2	0.9	0.9	0.25	0.01	0.26	1.3 (0.5)

a *The roto-translation parameters were calculated using the software SPM12*.

b *Mean absolute displacement and standard deviation (SD) values were calculated using the Optical Flow algorithm (RealTITracker toolbox)*.

For brain MRI, the standard and the proposed setups had comparable repositioning accuracy. The average absolute displacement was about 1 mm with a standard deviation of 0.5 mm.

For HN MRI, the standard RT-HN presented an average inter-fraction absolute displacement of about 1 mm (standard deviation 0.5 mm). When the proposed RT-B was used for HN MRI, the average inter-fraction absolute displacement was larger than 3 mm (standard deviation 1 mm), especially in the shoulders region (Supplementary Figure 2, middle row). In contrast, when the proposed RT-HN was used, i.e., including the shoulders in the immobilization mask, the average inter-fraction absolute displacement in the shoulder was highly reduced, and the values became comparable to the standard RT-HN.

## Discussion

In this work, we proposed a new immobilization setup for brain and HN RT consisting of a redesigned fixation system that can be combined with the diagnostic MRI H/HN coils. The presented results demonstrate the feasibility of integrating a fixation mask in the H/HN coils. The proposed setup allows several important advantages: (1) acquisition of MR images with diagnostic quality in TP, (2) high SNR, (3) homogenous signal for a relatively large field of view, (4) restricted motion (about 1 mm), (5) reproducibility of subject repositioning. These advantages altogether with the simplicity of assembling the presented setup, which uses widely available diagnostic coils and well validated immobilization masks (18, 21, 22), demonstrate the added practical value of the presented solution compared to the state-of-the-art.

From the presented results, we observe that the three-points head-mask is sufficient for accurate inter-fraction patient repositioning for MRI brain RT (average subject movement about 1 mm). This is comparable to the other state of the art setups (7–12). For HN RT, the presented results on one volunteer indicate that a three-points mask extended to the shoulders may be needed to reduce motion especially in the shoulder region. However, this cannot be considered as a general conclusion for HN. For the performed experiments, a standard neck support was used. The inter-fraction repositioning accuracy can be further improved if individualized supports are adopted (18).

The compatibility of the presented setup with the standard MRI table top and clinical diagnostic H/HN coils also allows for a reduction of the MRI examination time and the related costs; about 5 min are needed for table top replacement and fixation of the flexible surface coils. The presence of more receive elements in the H/HN MR coils, compared to surface flexible loop coils, facilitates higher acceleration in parallel imaging, leading to an additional reduction of examination time, which is important for MR acquisitions sensitive to motion. Moreover, as shown in Ruytenberg et al. (9), issues related to the use of surface flexible coils, such as coupling, malfunctioning of the connectors, and technicians dependent coil positioning, are avoided.

Surface flexible coils allow sufficient image quality only below the coils, but lead to poor SNR and thus poor image quality and reproducibility in regions away from the coils. Our solution allows for high SNR and diagnostic image quality on a large field of view, as shown in Figure 2. The aforementioned advantages are relevant for MRI acquisitions demanding high SNR or that are very sensitive to motion artifacts, such as FLAIR or T2-weighted Spin Echo images. This might be particularly beneficial for RT-HN treatments, where a large field of view needs to be imaged with diagnostic quality.

In this work, MR images were acquired only on two volunteers. We acknowledge that this may be seen as a possible limitation. However, we believe that the main conclusion of this work, i.e., the significant increase of SNR in a large field of view allowing diagnostic image quality in TP, is supported by our findings and valuable to be reported. Additionally, given the minimal modifications to the basic structure/geometry of the immobilization mask, motion restriction and repositioning accuracy could be expected to be equivalent to the existing mask system. This is confirmed by our results, which serve more as an example of using the new mask than as a quantitative study. Still, we do not see any fundamental differences or practical hurdles hindering clinical translation. Of course, a clinical study needs to be performed on patients to confirm the presented results in clinical settings.

For the clinical use, a robust construction of the thermoplastic mask holder has to be created (e.g., by 3D printing) and then further validated to guarantee the same patient positioning on the MRI and irradiation tables. In particular, for the irradiation tables, the base of the clinical MRI head coil will be replaced with radiolucent components. The use of different materials to construct the MRI and the irradiation thermoplastic holders can in principle allow maximization of radiofrequency transparency for the MRI, and radiolucency for RT treatments, respectively.

In a combined CT-MRI workflow, one could consider performing diagnostic MRI scanning not only in TP, and rely on rigid registration to a CT scan acquired in TP. This allows high quality MR images to be used for delineation. However, registration errors will remain, particularly for 2D MR images with relatively thick slices. For these acquisitions, the transfer of information derived from MR images to CT will be sub-optimal. Furthermore, motion artifacts are more likely to occur for MRI acquisitions without immobilization masks.

The proposed setup is particularly attractive for MR-only radiotherapy, where MR scanning has to occur in TP. By exploiting the diagnostic MRI image quality in TP, achievable with the proposed setup and the possibility to generate reliable synthetic CT images from these MRI images for dose calculations (23–26), the proposed setup represents an excellent platform to implement an MRI-only workflow for brain and HN RT (16, 27–29). There will be no need to perform MRI scans during RT treatment planning with different setups (i.e., a diagnostic MRI and an MRI in TP) and, more importantly, additional radioactive dose due to planning CT scans being avoided (7). This will lead to a more efficient workflow with a reduced number of examinations and an increased patient comfort. However, the use of synthetic CT images generated from MR images acquired on the MR table need to be verified for the Linac tables.

Instead, position verification would not be a problem if such a setup would be adopted for the MR-Linac, since diagnostic MR, treatment planning, and irradiations would be performed without the need of repositioning the patient among different machines. This setup, combined with MR-Linac systems and synthetic CT generation in MR-only workflows, will allow for online treatment re-planning on daily anatomy based on diagnostic quality MR images acquired in TP. Of course, as aforementioned, radiolucent materials will have to be adopted for the construction of the coil, and the impact of such a setup on the delivered dose will need to be assessed.

Finally, it is worth mentioning that this setup is directly applicable to both 1.5 T and 3 T scanners given the same receive H/HN coil geometry. Provided minor modifications, it can be applied to different head coils and MRI tables, e.g., flat table tops. This proves the high level of transferability of the presented setup. Outside RT settings, this solution can also be exploited for diagnostic MRI, especially 3D acquisitions regularly suffering from artifacts due to head motion (30). This is particularly relevant for clinical radiological settings where non-cooperative patients are scanned. Finally, we believe that MRI applications requiring high resolution 3D isotropic scans, such as vessel wall imaging, which are very sensitive to motion artifacts due to their long acquisition time, could benefit from the proposed setup (31, 32).

## Conclusions

We presented a new immobilization setup for brain and head-and-neck MR imaging in radiotherapy treatment position. This setup fits in the clinically diagnostic MRI head and head-and-neck coils allowing: superior diagnostic MRI image quality in treatment position, high SNR images, accurate subject repositioning, and motion restriction.

## Data Availability

Datasets are available on request: the MRI data supporting the conclusions of this manuscript will be made available by the authors, without undue reservation, to any qualified researcher.

## Ethics Statement

This study was carried out in accordance with the recommendations of the Medical Ethics Committee of the University Medical Center Utrecht. The protocol was approved by the Medical Ethics Committee of the University Medical Center Utrecht. All subjects gave written informed consent in accordance with the Declaration of Helsinki.

## Author Contributions

SM, FD, and RN performed the MR scans. All the authors contributed to the scientific debates and writing the manuscript.

### Conflict of Interest Statement

The authors declare that the research was conducted in the absence of any commercial or financial relationships that could be construed as a potential conflict of interest.
