# Supplementary material for: Brain and Head-and-Neck MRI in Immobilization Mask: A Practical Solution for MR-Only Radiotherapy
Source: Front Oncol. 2019 Jul 17;9:647. doi: 10.3389/fonc.2019.00647 (PMC6650525; doi:10.3389/fonc.2019.00647)
Supplement: Supplementary file 1 [file Data_Sheet_1.docx]

Supplementary Material

# Supplementary Figures and Tables

## Supplementary Figures

**Supplementary Table 1.** For each test: summary of the performed MRI sequences and sequence parameters.

| **Tests** | **Region** | **Sequence** | **Parameters** |
| --- | --- | --- | --- |
| **SNR** | Brain/HN | 2D T1-weighted Incoherent Gradient Echo (RF spoiled) | **Transversal and Sagittal**, FOV=230x230x6mm^3^, voxel size=1.8x1.8x6mm^3^, FA=8º, TR=4.5ms, TE=2.2ms, dynamics=2, scan time=1sec. |
| **Image Quality** | Brain | 3D T1-weighted Ultrafast Gradient Echo | **Transversal**, FOV=230x230x120mm^3^, voxel size=0.96x0.96x1mm^3^, FA=8º, TR=8.1ms, TE=3.7ms, TFE=224, SENSE=2(RL), scan time=2:49min. |
|  |  | 3D T2-weighted Turbo Spin Echo FLAIR | **Transversal**, FOV=230x230x120mm^3^, voxel size=0.96x0.96x0.60mm^3^, TR=4800ms, TE=326ms, TI=1650ms, TSE=199, averages=2, SENSE=3(RL), SENSE=1.2(FH), scan time=5:02min. |
|  |  | 2D T2-weighted Turbo Spin Echo | **Transversal**, FOV=230x230x120mm^3^, voxel size=0.45x0.45x4mm^3^, FA = 90º, TR=3396ms, TE=80ms, TSE=15, SENSE=1.2(RL), scan time=2:02min. |
|  | HN | 2D T1-weighted Turbo Spin Echo | **Transversal**, FOV=280x201x120mm^3^, voxel size=0.73x0.73x2mm^3^, FA=90º, TR=678ms, TE=6.1ms, TSE=10, averages=2, SENSE=2(RL), scan time=2:39min. |
|  |  | 2D T2-weighted Turbo Spin Echo mDIXON | **Transversal**, FOV=450x278x249mm^3^, voxel size=0.94x0.94x3mm^3^, FA=90º, TR=9023ms, TE=100ms, ΔTE=1ms, TSE=27, averages=2, SENSE=2(AP), scan time=5:25min. |
| **Motion Restriction** | Brain/HN | 2D cine-MR T1-weighted balanced Gradient Echo | **Sagittal,** FOV=400x250x10mm^3^, voxel size=1.4x1.4x10mm^3^, FA=4º, TR=3.1ms, TE=1.4ms, dynamics=300, scan time=1:22min.  **Transversal**, FOV=250x176x10mm^3^, voxel size=1.4x1.4x10mm^3^, FA=4º, TR=3.2ms, TE=1.5ms, dynamics=300, scan time=0:58min. |
| **Inter-fraction Repositioning** | Brain | 3D T1-weighted Ultrafast Gradient Echo | **Transversal**, FOV=230x230x120mm^3^, voxel size=0.96x0.96x1mm^3^, FA=8º, TR=6.3ms, TE=2.8ms, TFE=224, SENSE=2(RL), scan time=1:40min. |
|  | HN | 3D T1-weighted Ultrafast Gradient Echo | **Transversal**, FOV=230x230x120mm^3^, voxel size=0.96x0.96x1mm^3^, FA=16º, halfscan=0.625, TR=3.9ms, TE=1.7ms, averages=2, SENSE=1.5(AP), scan time=1:30min. |


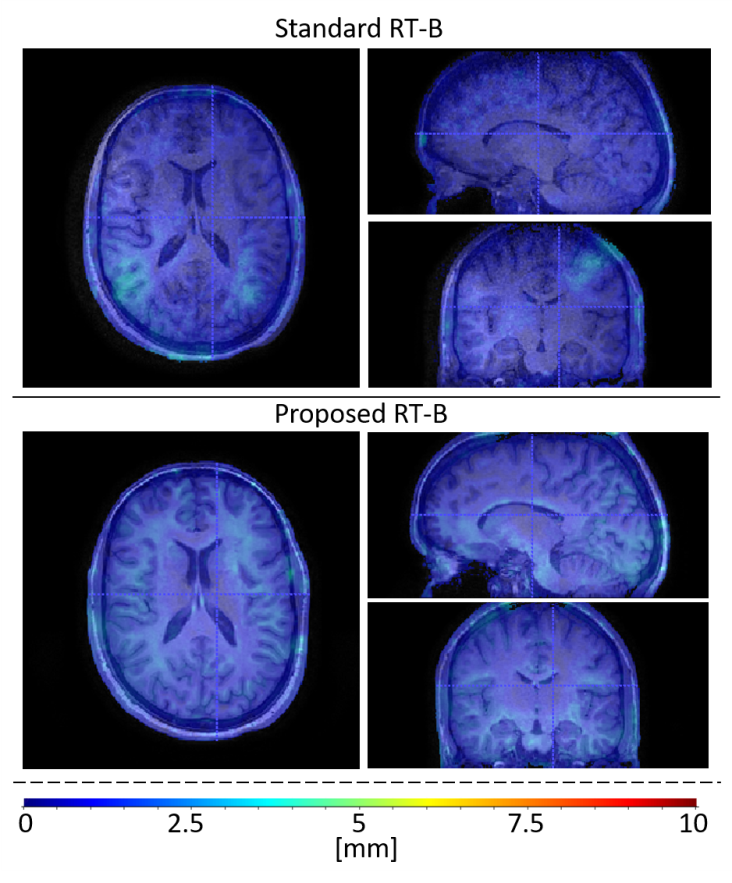


**Supplementary Figure 1.** BRAIN: Inter-fraction absolute displacement maps for the standard and the proposed RT-B setups. These maps show that the standard and the proposed RT-B setups have comparable inter-fraction repositioning accuracy (see **Table 1** in the manuscript for quantitative evaluation).


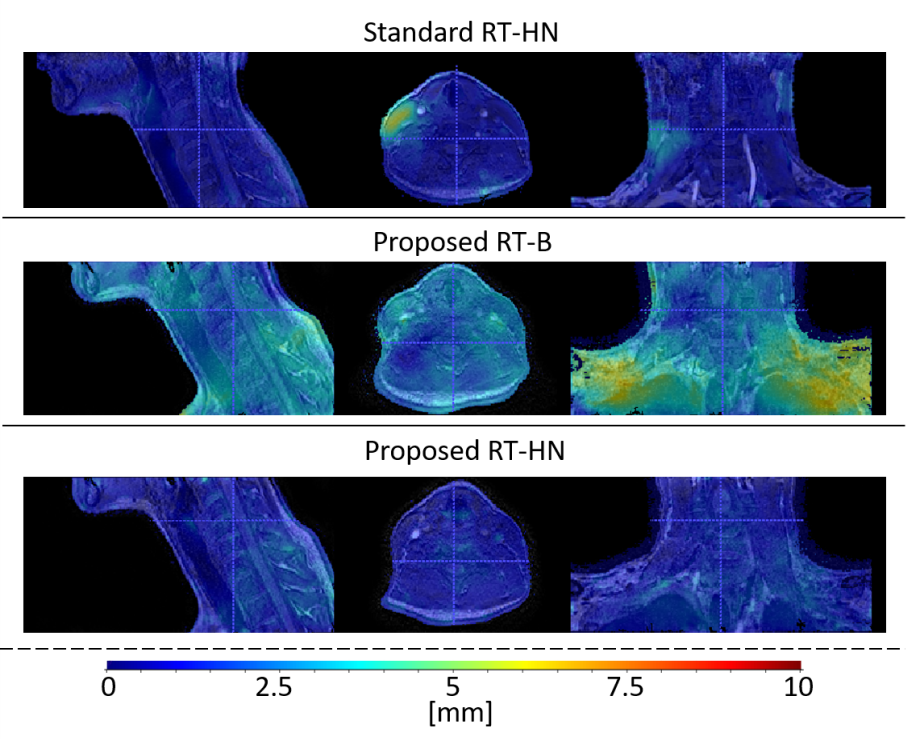


**Supplementary Figure 2.** HEAD-AND-NECK: Inter-fraction absolute displacement maps for the standard RT-HN setup, the proposed RT-B setup (three-points head-mask), and proposed RT-HN setup (three-points head-and-shoulder mask). If the proposed RT-B setup (middle row) is used for HN MRI, the inter-fraction absolute displacement is higher than the inter-fraction absolute displacement observed for the standard RT-HN setup (top row), especially around the shoulders. If the immobilization mask is extended to the shoulders, i.e. the proposed RT-HN setup is used (bottom row), the inter-fraction absolute displacement is highly reduced: mean inter-fraction absolute displacement and standard deviation values are comparable to the standard RT-HN setup (top row).

**
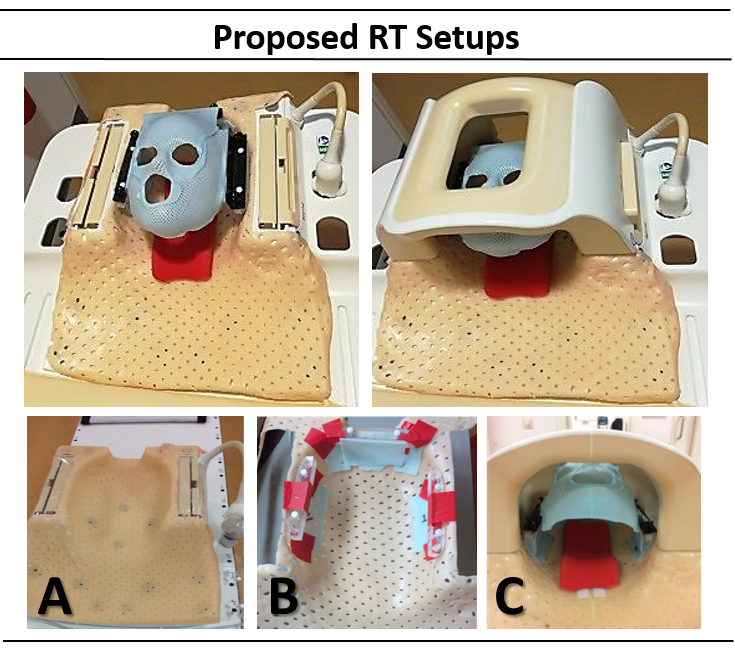
**

**Supplementary Figure 3.** The proposed RT setup consists of an in-house thermoplastic base, which is molded to perfectly fit the base of the clinical head coil (**A**). The anchor points for the immobilization masks are attached to this base (**B**). The immobilization mask is fixated to these points and fits inside the clinical head coil (**C**). An individualized neck support is also fixated to the thermoplastic base.
